# An insight into the gene expression evolution in *Gossypium* species based on the leaf transcriptomes

**DOI:** 10.1186/s12864-024-10091-x

**Published:** 2024-02-14

**Authors:** Yuqing Wu, Rongnan Sun, Tong Huan, Yanyan Zhao, Dongliang Yu, Yuqiang Sun

**Affiliations:** College of Life Sciences and Medicine, Zhejiang Sci-Tech University, Hangzhou, 310018 China

**Keywords:** Gossypium, Transcriptome, Expression evolution, Orthologous groups

## Abstract

**Background:**

Gene expression pattern is associated with biological phenotype and is widely used in exploring gene functions. Its evolution is also crucial in understanding species speciation and divergence. The genus Gossypium is a bona fide model for studying plant evolution and polyploidization. However, the evolution of gene expression during cotton species divergence has yet to be extensively discussed.

**Results:**

Based on the seedling leaf transcriptomes, this work analyzed the transcriptomic content and expression patterns across eight cotton species, including six diploids and two natural tetraploids. Our findings indicate that, while the biological function of these cotton transcriptomes remains largely conserved, there has been significant variation in transcriptomic content during species divergence. Furthermore, we conducted a comprehensive analysis of expression distances across cotton species. This analysis lends further support to the use of *G. arboreum* as a substitute for the A-genome donor of natural cotton polyploids. Moreover, our research highlights the evolution of stress-responsive pathways, including hormone signaling, fatty acid degradation, and flavonoid biosynthesis. These processes appear to have evolved under lower selection pressures, presumably reflecting their critical role in the adaptations of the studied cotton species to diverse environments.

**Conclusions:**

In summary, this study provided insights into the gene expression variation within the genus Gossypium and identified essential genes/pathways whose expression evolution was closely associated with the evolution of cotton species. Furthermore, the method of characterizing genes and pathways under unexpected high or slow selection pressure can also serve as a new strategy for gene function exploration.

**Supplementary Information:**

The online version contains supplementary material available at 10.1186/s12864-024-10091-x.

## Introduction

The genus Gossypium harbors about 45 diploids that are cytogenetically clustered into eight genome groups (A-G and K) and seven allotetraploids [AD_1_ to AD_7_] that originated from hybridization between A- and D-genome diploids [1–4]. These species are diverse in geographical distribution, morphology, and fiber characteristics, thus providing an ideal system for understanding the mechanisms underlying species speciation, polyploidization, domestication, and adaptation.

Gene expression connects the genetic basis and molecular function. Taking advantage of the development of high-throughput techniques such as microarray and RNA sequencing (RNA-seq), genome-wide expression analysis has become a widely used tool in various fields of biological research [5]. In cotton species, differential gene expression (DGE) and co-expression analyses have identified numerous genes and networks involved in plant response to environmental stresses (*e.g.*, cold, drought, salinity, alkalinity, heavy metal, bollworm, and fungal pathogens) [6–10], fiber initiation and development [11–16], and morphology formation [17, 18].

Gene expression analysis has also been applied in deciphering the molecular mechanism underlying plant polyploidization. For example, the assessment of homoeolog expression bias (HEB) and expression level dominance (ELD) has greatly advanced our understanding of the re-assignment of biological functions in polyploids. HEB and ELD describe the relative expression levels between homoeologs (duplicate genes arose from polyploidization) and gene expression variation between the polyploid (hybrid) and progenitors, respectively [19]. Studies in the last two decades revealed that HEB and ELD are common features in natural and synthetic cotton polyploids via small- or large-scale methods of expression analysis (e.g., Real-time Quantitative PCR, single strand conformation polymorphism, mass spectrometry, microarray, and RNA-seq) [20–28]. These works also revealed that parental legacy was the leading cause for the expression patterns in polyploid cotton species, while long-term evolution after polyploidization was also involved in the modulation [24, 26–28].

In short, expression analysis has extensively promoted the understanding of cotton biology in various scenarios. However, study on gene expression evolution during cotton species speciation and evolution is still scarce, and some related issues remain to be discussed. First, comparative genomics analysis has revealed the extensive conservation of genetic basis across cotton species, such as the large-scale syntenic blocks between *G. arboreum* (A_2_) and *G. raimondii* (D_5_) (~ 80%), D_5_ and D_t1_ (the D-subgenome of AD_1_) (~ 90%), as well as A_2_ and *G. austral* (G_2_) (~ 70%) [29, 30]. In contrast, comparative transcriptomics analysis usually focuses on the DGE in particular cotton species under different conditions (e.g., stresses) and developmental stages or on a small group of species (e.g., diploid parents and polyploid progeny). In contrast, the transcriptomic content and their expression evolution have not been discussed in detail.

Next, although it is widely accepted that D_5_ is the most potential D-genome donor of the natural tetraploid cotton species, discussion about the A-genome donor has been continued for decades, and independent evidence supports A_2_ or A_1_ as the A-genome donor [31, 32]. The availability of the A_2_ and A_1_ genome sequences and comparative genomics analysis strongly indicated that it is either A_1_ or A_2_, but their common ancestor A_0_ gives rise to the A-genome of tetraploids [33]. Nevertheless, another question emerges accompanied by the gradual calm down of the debate, *i.e.*, as A_0_ is very possibly extinct, which one of A_1_ and A_2_ is a better substitute for A genome parent when estimating the expression change upon polyploidization (*e.g.*, ELD) in natural tetraploid cotton species?

To provide a more comprehensive understanding of the aforementioned concerns, we have conducted a comparative analysis of the transcriptomes of seedling leaves from six diploid and two natural tetraploid cotton species. By utilizing ortholog groups, we have also evaluated the expression evolution associated with cotton polyploidy.

## Results

### Comparative analysis of the gene content of the leaf transcriptomes

To include more cotton species in this study, we collected a series of public RNA-seq data in addition to the data generated in this work (RNA-seq of A_1_ seedling leaves). For a more accurate estimation of the gene expression levels, we only focused on the cotton species with well-established genome assemblies and comprehensive annotation. Moreover, we tried to assess the same tissues at the same period to minimize the systematic discrepancy. Finally, the RNA-seq data of seedling leaves from eight cotton species were collected, referring to the diploids of genome group A (A_1_ and A_2_), D (D_1_ and D_5_), E (E_1_), and G (G_2_), as well as the natural polyploids AD_1_ and AD_2_ (Table 1). The subgenomes of AD_1_ (A_t1_ and D_t1_) and AD_2_ (A_t2_ and D_t2_) were considered independent organisms during expression evolution analysis. The closely related species *Theobroma cacao* was used as the outgroup when performing phylogenetic analysis.

**Table 1:** Data source of the reference genomes and RNA-seq data

Group	Species	Genome version (Database)	RNA-seq data
Tetraploid	AD_2_	HAU_v2 (CottonGen)	SRR8089908; SRR8089972; SRR8089978
	AD_1_	NAU-NBI_v1.1 (CottonGen)	SRR8090032; SRR8090033; SRR8090035
Diploid	A_2_	WHU-updated v1 (CottonGen)	SRR13933601; RR13933602; SRR13933598
	G_2_	CRI_v1.1 (CottonGen)	SRR8694038; SRR8694039; SRR8694045
	D_5_	BGI-CGP-draft_v1 (CottonGen)	SRR8267559-SRR8267561
	A_1_	WHU_v1 (CottonGen)	SRR22211789-SRR22211791
	E_1_	GCA_020496765.1 (NCBI)	SRR13933592-SRR13933594
	D_1_	CRI_v1 (CottonGen)	SRR8267613-SRR8267615
Outgroup	*T. cacao*	cocoa_v2 (MaGenDB)	SRR851884; SRR851885

**Fig. 1:**
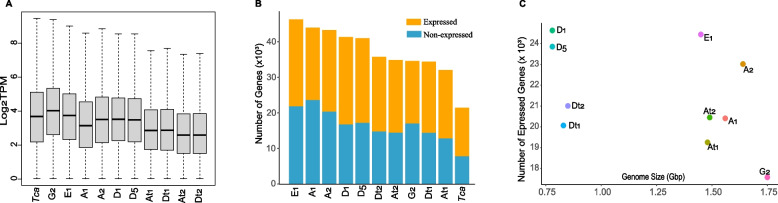
Statistics on expressed coding genes in the seedling leaves of cotton species. **A** Distribution of expression levels of coding genes; **B** Number of expressed genes; **C** Relationship between the number of expressed genes and genome size. A_t1_/A_t2_ and D_t1_/D_t2_ indicate the A- and D-subgenome of the tetraploids AD_1_/AD_2_, respectively

According to the genome annotation, about 32,000 to 46,200 coding genes are identified in cotton species (or subgenomes). Herein, RNA-seq data analysis was performed to estimate the expression levels of these genes in the seedling leaves. About 18 to 32 million short reads were aligned to the reference cotton genomes, with an overall mapping ratio of about 95% (Table S1). Most of the genes expressed at the levels of log_2_TPM ranged from about 2 to 4. Overall, the AD_1_ and AD_2_ subgenomes encoded genes were expressed at lower levels than those of diploids (Fig. 1A).

Under the cutoff of average TPM ≥ 1, we found that about 46.4 ~ 60.1% of the cotton coding genes were expressed (Fig. 1B). Notably, the number of expressed genes was not correlated with the genome size or total gene number (Fig. 1C). For example, the largest and smallest number of expressed genes were detected in D_1_ and G_2_, respectively, whereas the genome size of D_1_ (~ 0.78 G bp) was much smaller than that of G_2_ (~ 1.75 G bp). Besides, the number of expressed genes does not always correlate with the phylogenetic relationship. For instance, despite the similarity in the number and ratio of expressed genes in the D-genome species D_1_ (24,631) and D_5_ (23,853), we observed that the number of expressed genes in A_2_ (23,026) was closer to that of D_5_ but much greater that of another A-genome species A_1_ (20,407). In addition, a comparable number of A_t_ and D_t_ encoded genes were expressed (~ 20,000) in AD_1_ and AD_2_, which was approximate with the number of expressed genes in A_1_ but much smaller than in D-genome species.

As the extremely low-expressed genes were excluded from this analysis, we acknowledge the possibility that this may have led to deviated statistics. Therefore, we used the cutoff of average TPM ≥ 0.1 and repeated the calculation of the expressed genes in cotton leaves. We found that the ranking of the cotton species was almost identical to the results mentioned above when lowly-expressed genes were included in the scope of the investigation (Figure S1). Furthermore, to assess the potential impact of sequencing depth on the number of expressed genes detected, we investigated the accumulation of detected genes as the depth of RNA-seq data increases. The results indicate that the current datasets were sufficient to identify nearly all expressed genes in the investigated species (Figure S2). All these findings suggest that species that are closely related in phylogeny may not necessarily exhibit similar patterns in the number of expressed genes.

**Fig. 2:**
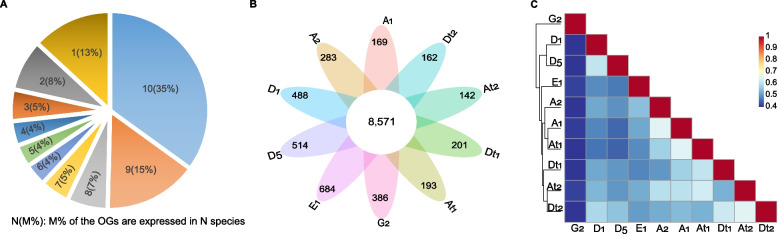
Comparative analysis of expressed orthologous genes across cotton species. **A** Statistics on the number of species in which the orthologous groups (OGs) are expressed. An OG is marked as expressed if one or more of its gene components show detectable expression (average TPM ≥ 1); **B** Statistics on conserved and species-specific OGs; **C** The relationship among cotton species estimated by OG expression profile. A binary “0/1” matrix was constructed to represent OG expression (0: not expressed; 1: expressed), which was then used to calculate pairwise Pearson correlation coefficients

We also constructed orthologous groups (OGs) to evaluate the differences in gene content across the cotton transcriptomes, in order to minimize the deviation caused by genome assembly and annotation. OrthoFinder generated 34,456 OGs across ten cotton species (subgenomes) and the outgroup *T. cacao* (Table S2), which contained about 97% of the annotated coding genes. 24,517 OGs were expressed in at least one species (subgenomes), including about 87% in two or more species. 35% of these OGs (8,571) were expressed in all cotton species. In contrast, only about 1 ~ 3% of these OGs were expressed specifically, with the number ranging from 142 in A_t_ of AD_2_ to 684 in E_1_ (Fig. 2A and B). Notably, for the 8,571 OGs expressed in all cotton species, 7,220 were detected in the *T. cacao* transcriptome, suggesting this gene set was conserved in evolution.

We assessed the relationship between phylogeny and transcriptomic content as well. Pairwise analysis revealed that cotton species shared about 68 ~ 94% of the expressed OGs. Unexpectedly, we observed that the species with closer phylogeny were not always more similar in terms of transcriptomic content. For instance, A_2_ shared more expressed OGs with D_1_, D_5_, and E_1_ than with A_1_ (Table S3), and A_t2_ showed a more similar set of OGs to the D_t_ genomes in tetraploids, but not to A_t1_, A_1_ or A_2_ (Fig. 2C).

### Similarity and evolution distance between gene expression patterns in cotton leaves

We then analyzed the evolution of gene expression patterns using the conserved genes for further characterization of the expression variation during cotton species divergence. OrthoFinder generated 11,627 species-complete OGs across ten cotton species (subgenomes) and the outgroup. After trimming the ambiguous and redundant copies, 7,668 1:1 OGs were generated to represent the conserved gene content across investigated species. To minimize the errors arising from the lowly expressed genes, we removed OGs with their components lowly expressed (average TPM < 1) in all species and finally acquired 5,963 OGs for subsequent analyses.

**Fig. 3:**
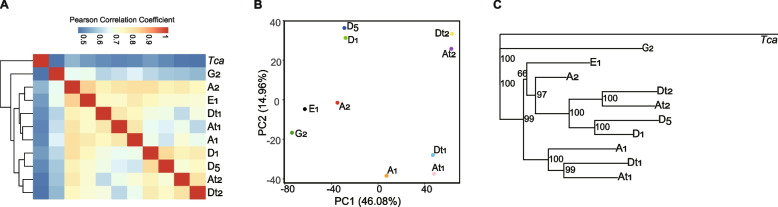
Comparative and phylogenetic analysis of the expression patterns of cotton species using the expression matrix of conserved genes. **A** Hierarchical clustering analysis of the expression patterns; **B** Principal Components Analysis of the expression patterns; **C** The expression tree of cotton species that was constructed based on the expression distance

We first analyzed the similarities of expression patterns between cotton species based on the pairwise Pearson correlation coefficients between the OGs (Fig. 3A). As a result, we found that the gene expression pattern in the outgroup *T. cacao* was significantly different from the cotton species, and G_2_ was relatively more distinct from others within the genus *Gossypium*. Consistent with the content analysis, we found that species from the same genome group were not always more similar in expression pattern. For example, though two diploid D-genome species (D_1_ and D_5_) were clustered together after hierarchical clustering, A_2_ was closer to E_1_ but not A_1_ in the cluster dendrogram. In addition, A_t_ subgenomes of AD_1_ and AD_2_ were more similar to each other than to their ancestor (D_5_) or ancestor-sisters (A_1_/A_2_). We also found that the expression pattern of A_1_ was more similar to the A_t_ in AD_1_, while the expression pattern of A_2_ was more similar to the A_t_ in AD_2_. PCA analysis also supported these phenomena (Fig. 3B), which revealed that the subgenomes of AD _1_ and AD _2_ showed different expression patterns from the other species according to PC1.

Then, we calculated the pairwise expression distance between designated species under the stationary Ornstein–Uhlenbeck model. The derived expression character tree indicated a similar relationship among species depicted by hierarchical clustering analysis (Fig. 3C), *i.e.*, the expression patterns between A_t_ and D_t_ were closer to each other than to other species. In addition, expression patterns of the subgenomes of AD_1_ were closer to A_1_, while AD _2_ subgenomes were closer to the A_2_ and D_1_/D_5_ clades.

### Analysis of expression change upon cotton polyploidization using different substitutes of At-genome donor

Due to the extinction of the A-genome donor (A_0_) of natural tetraploid cotton species, A_2_ is now commonly used as a substitute when analyzing the gene expression change upon polyploidization. As is revealed above, the gene expression patterns of the A_t_ subgenomes of AD_1_ and AD_2_ were closer to A_1_ and A_2_, respectively. Therefore, it is necessary and interesting to find out the deviations caused by the use of substitutes when analyzing the expression change upon polyploidization.

**Fig. 4:**
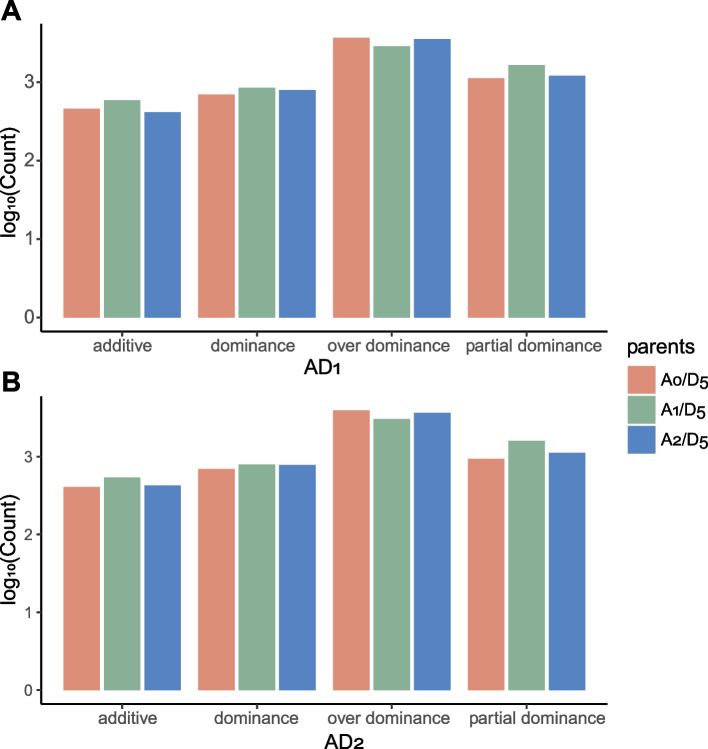
Expression change analysis upon cotton polyploidization. To assess the expression change in the polyploid progenies AD_1_ and AD_2,_ D_5_ was used as the D-genome parent, and A_1_, A_2_, and the inferred ancestor A_0_ were used as the A-genome parent, respectively. The expression pattern was determined based on the dominant/additive ratio

We first inferred the ancestor (A_0_) transcriptome of A_1_ and A_2_ based on the expression tree. Then, we compared the result of expression change analysis when using A_0_, A_1_, and A_2_ as the A-genome donor, respectively. Notably, as the gene expression levels in A_0_ were inferred based on the average TPM and no biological replicate was available, the MPV (mid-parent value) instead of the DEG-calling-based method (Rapp, 2019) was used for expression pattern binning. When using A_0_ and A_2_ as the parental species, fewer genes in AD_1_ were classified into the additive, partial dominance, and dominance groups, and more genes were classified into the over-dominance group (Fig. 4). For example, when A_0_ and A_2_ were used as the A-genome donors, 406 and 425 additively expressed genes were identified in AD_1_, which were significantly lower than the number (536) when A_1_ was used (χ^2^-test, *p*-value < 0.05). Similar results were observed when analyzing the expression change in AD_2_, indicating that the estimation of additive expression in natural tetraploid cotton species can be reasonably accurate when A_2_ was used as the parent species, but might be overestimated when A_1_ is used.

### Comparison of the expression evolving rate between progenitors and polyploid cotton species

To deepen our understanding of expression evolution after cotton species polyploidization, we compared the evolving rate of gene expression between two cultivated tetraploid cotton species (AD_1_ and AD_2_) and their progenitors (A_2_ and D_5_). The gene were first grouped into 102 sets according to the KEGG pathway annotation, and the sets containing more than ten gene components were compared between species.

The gene expression in several pathways showed significant variation (Table 2), referring to the housekeeping functions such as nucleotide and amino acid metabolism, mismatch repair, carbohydrate metabolism, and circadian rhythm. Notably, the expression of the genes involved in the pathway ‘Plant hormone signal transduction’ evolved faster in A_2_ than in AD_1_ and AD_2_. In the investigated gene set, there were twelve gene components in the corresponding gene set of this pathway, such as phytochrome-interacting factor 3 (PIF3), auxin-responsive proteins (IAA and GH3), regulatory protein NPR1, protein phosphatase 2C (PP2C), and jasmonate ZIM domain-containing protein (JAZ). In addition, a comparison between the tetraploids revealed that the expression of pathway ‘Fatty acid degradation’ evolved significantly faster in AD_1_ than in AD_2_, with the gene components including alcohol/aldehyde dehydrogenase, acyl-CoA oxidase, and long-chain acyl-CoA synthetase.

**Table 2:** Comparison of the evolution rate of KEGG pathways between diploid parents and polyploid cotton species

Groups	Pathways	*p*-value
AD_1_ < A_2_^a^	Plant hormone signal transduction	7.79E-04
	Pyrimidine metabolism	2.16E-09
AD_2_ < A_2_	Plant hormone signal transduction	4.15E-06
AD_1_ < D_5_	Biosynthesis of secondary metabolites	3.17E-11
	Pentose phosphate pathway	3.32E-04
AD_2_ < D_5_	Circadian rhythm	7.13E-09
AD_1_ > A_2_	Ribosome biogenesis in eukaryotes	3.78E-04
AD_2_ > A_2_	Mismatch repair	4.07E-06
	Glycine, serine and threonine metabolism	1.37E-04
AD_1_ > AD_2_	Fatty acid degradation	3.64E-05

^a^The symbol ‘ > ’ or ‘ < ’ indicates greater or smaller evolution rate

The results from comparative analysis of the evolution rate suggest that gene expression has not undergone extensive modulation following cotton polyploidy. This is supported by the observation that only a few pathways in the polyploid progenies exhibited significantly greater or less rate compared to the diploid ancestors. Furthermore, rare variations were observed between AD_1_ and AD_2_ in the evolution rate for designated pathways, indicating that a limited range of expression modulation occurred during their relative short history of independent evolution.

### Gene expression conservation within the genus *Gossypium*

We analyzed the conservation of gene expression within the genus Gossypium at the level of a single gene by estimating the selection pressure (*w*). The selection pressure for the 5,963 investigated genes ranged from 0.01 to 1.23, with 5% of the genes under the pressures of > 0.80 (genes under high selection pressure, HSG), and < 0.11 (genes under low selection pressure, LSG), respectively. Two genes encoding Elongator complex protein 3 (ELP3) and Plastid Movement Impaired 1-Related 1 (PMIR1) under the highest selection pressure (*w* ≈ 1.23), while the genes encoding photosynthesis-related proteins Photosystem I light harvesting complex gene 2 (LHCA2) and starch synthase were under the lowest selection pressures (*w* ≈ 0.02). GO enrichment analysis did not show enriched LSGs or HSGs in any biological process. Interestingly, the enrichment analysis revealed that the chloroplast-located proteins were over-represented in the LSGs (elimKS: 4.20E-5) but under-represented in the HSGs (elimKS: 1.10E-11).

**Fig. 5:**
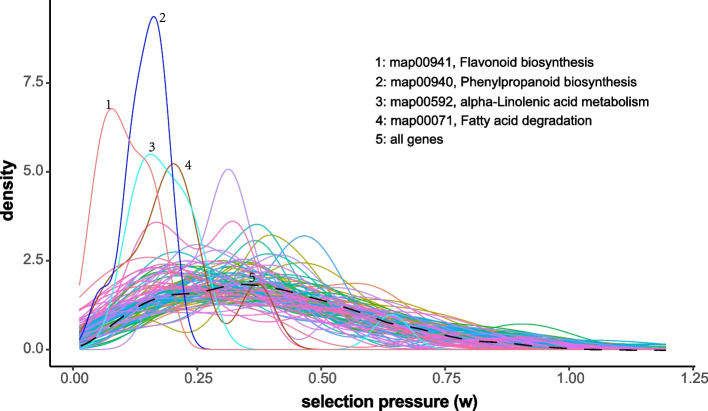
Distribution of selection pressures on cotton genes in different biological pathways. Genes conserved in primary sequence and expressed across all examined cotton species were categorized into distinct biological pathways using KEGG annotation. The selection pressure (*w*) for these genes was determined using Bayes’ theorem and a TPM matrix depicting their expression in seedling leaves of different species. Each curve represents the distribution of *w* for genes within a specific KEGG pathway, while the dashed line denotes the overall distribution of *w* for all conserved genes

Moreover, we assessed the variation of expression selection pressures among different KEGG pathways. We found that gene components of some pathways were more frequently under low selection pressures, such as the biosynthesis of secondary metabolites (*e.g.*, Flavonoid biosynthesis and Phenylpropanoid biosynthesis) and lipid metabolism (*e.g.*, alpha-Linolenic acid metabolism and Fatty acid degradation) (Fig. 5). In contrast, few pathways contained remarkably abundant HSGs. Therefore, we checked the pathways under the highest average selection pressures and found that they were mainly associated with the housekeeping functions such as protein biosynthesis, folding, sorting, and degradation (*e.g.*, Aminoacyl-tRNA biosynthesis, Proteasome, and Nucleocytoplasmic transport), Glycan biosynthesis (*e.g.*, GPI-anchor biosynthesis and N-glycan biosynthesis), transcription regulation (*e.g.*, Basal transcription factors and Spliceosome), Cell motility and growth (*e.g.*, Regulation of actin cytoskeleton and Cell cycle) (Table S4).

## Discussion

Gene expression patterns vary on tissues, developmental stages, and environmental stresses and have been widely used and are still crucial for gene function exploration. Cotton species are globally important economic crops. In the last decades, especially after the availability of a growing number of cotton genomes, high-throughput expression analysis has been extensively applied to understand the molecular and cellular processes underlying cotton development and stress response. Nevertheless, these studies mainly focused on the differential gene expression of single species under various conditions or development stages. Although the expression changes between the diploid parents and polyploid progenies has also been analyzed for unraveling the mechanisms of function re-assignment in subgenomes, overall, limited attention has been paid to the expression change or its role in a broader range of cotton species in evolutionary scenarios.

### Expression patterns in seedling leaves are extensively varied across cotton species

In the present work, we comparatively analyzed the gene expression patterns in the seedling leaves of several cotton species and assessed their divergence during evolution. We observed significant differences in the number of expressed genes across species. However, it remains uncertain whether the variable quality of genome assembly and annotation has significantly influenced this phenomenon. Therefore, we refrain from making arbitrary inferences based solely on the number of expressed genes. Instead, we further compare the gene content of the transcriptomes according to the orthologs groups. Interestingly, very few OGs (< 4%) were species-specifically expressed, indicating that the cellular biological processes in the leaves are conserved within the genus Gossypium and that gene gain/loss during species divergence might have played important roles in the construction of the transcriptomes and the formation of different phenotypes of cotton leaves.

Polyploidization is a common phenomenon in eukaryotic organisms and plays a crucial in species speciation and evolution [34–36]. After genome merge and doubling, expression modulation is essential for maintaining genome stability and forming novel phenotypes in new polyploids. This work revealed that the phylogenetically related species mainly shared a more similar profile of expressed OGs, such as the groups of D_1_/D_5_ and E_1_/A_1_/A_2_/A_t1_. However, polyploidization and subsequent evolution may have significantly altered the gene expression in the subgenomes of AD_1_ and AD_2_, as the profiles of expressed OGs in D_t_ were more similar to A genomes than D genomes.

### Expression modulation after polyploidization altered the expression patterns of subgenomes

HEB and ELD are widely used to describe the expression changes in polyploids and have revealed some important mechanisms underlying the environmental stress response of polyploid organisms. For example, HEB in the tetraploid coffee and cotton species is closely associated with temperature and salt conditions [28, 37–39], respectively. Unfortunately, the ELD analysis in natural tetraploid cotton species inevitably faces the problem of the extinction of the A-genome ancestor. A_2_ is now usually used as the substitute for A_t_-genome donor, but if its use will and to what extent impair the accuracy of expression change analysis has been rarely discussed.

Based on the similarity and expression distance analyses, this work revealed that the gene expression pattern in A_2_ was more closely related to the A genome ancestor. The additive expression was more accurately estimated when A_2_ rather than A_1_ was used to analyze the gene expression change in AD_1_ and AD_2_. Therefore, in addition to the evidence from phylogeny, expression evolution analysis also indicated that A_2_ is a reasonable substitute A-genome donor for expression change analysis (*e.g.*, ELD) in natural polyploid cotton species.

Besides, this work revealed that the expression patterns of AD_1_ and AD_2_ subgenomes were closely related to A-genome and D-genome diploid cotton species, respectively (Fig. 3C). This phenomenon was similar to a previous proteomics analysis in cotton fiber, which showed that the fiber proteomes of AD_1_ and AD_2_ were closely related to the parental A- and D-genomes, respectively [40]. These facts indicated that, although they originated from a common ancestor and conserved in genetic basis and even transcriptomic content, independent evolutionary history has extensively modulated the gene expression patterns of the sub-genomes in natural cotton polyploids. Moreover, the diverged expression patterns indicated the inter-subgenome transcriptional regulation commonly worked there in cotton polyploids, and the dominant regulatory subgenomes might differ between AD_1_ and AD_2_, at least in the seedling leaves.

### Expression modulation of some stress responsive pathways might be crucial for the divergence of cotton species

Further analysis showed that few pathways were different in expression evolving rate between diploid parents (A_2_ and D_5_) and progenies (AD_1_ and AD_2_) or between the progenies, and the pathways associated with the leaf-specific physiological roles (e.g., photosynthesis) were not significantly varied, suggesting either polyploidization or independent evolution of AD_1_ and AD_2_ have dramatically altered cellular functions. Nevertheless, expression of the genes in hormone mediated signal transduction pathway evolved remarkably faster in the parent species A_2_ than in the progenies. Plant hormone signal transduction plays diverse biological roles in plant seedling germination, growth, fruit ripening, leaf senescence, and stress response [41, 42]. A faster expression evolving rate of hormone signaling might point to a broader range of adaptational changes of transcriptional regulation in A_2_, while gene duplication arose by polyploidization might have made the tetraploids more flexible for evolutionary adaptation.

In addition, although extensive variation between AD_1_ and AD_2_ in the leaf morphology and physiology are observed, such as leaf thickness, area, chlorophyll content, and photosynthetic rate [43], this work revealed that only the pathway of fatty acid degradation was significantly varied between them in the evolving rate of gene expression. This was congruent with the subsequent finding that expression of fatty acid degradation pathway tended to undergo a lower selection pressure within the genus Gossypium. Fatty acid degradation in plants provides the energy and carbon source for diverse processes, and the activity of its components is often associated with stress response. For cotton species, Guo et al*.* revealed that the majority of aldehyde dehydrogenases in A_2_ and AD_1_ were upregulated under the conditions of high salinity and drought [44], while Dong et al*.* and Tian et al. showed that acyl-CoA oxidases in AD_1_ were responsible for various stresses, such as high-temperature, low-temperature, salt, and simulated drought stress [45, 46]. It is reasonable that its diverged expression pattern in cotton species will induce large-scale adjustment in cellular process and function. Nevertheless, further study is to be expected for the exploration of the biological roles of fatty acid degradation related genes in the morphology and physiology of cotton leaves.

Notably, apart from fatty acid degradation, we found that flavonoid biosynthesis related pathways were also under low selection pressure during cotton species evolution. Flavonoids are ubiquitously involved in plant tissue coloration and help plant development and growth under various biotic and abiotic stresses [47]. For cotton species, flavonoids and derivatives are the main biochemical basis for the coloration of leaf and fiber [48, 49], and affect cotton growth, development, and defense against the stresses like ultraviolet radiation and *Verticillium dahliae* [50]. The composition of flavonoids is diverse in plants, including cotton species, such as recent studies identified 122 and 190 flavonoids in A_2_ petals and AD_1_/AD_2_ leaves, respectively [50, 51]. Thus, a low level of selection pressures for the flavonoid biosynthetic genes is probably an evolutionary consequence of the requirement of diverse flavonoid profiles during cotton species adaptation to their environments.

In contrast, this work also identified that the genes such as ELP3 and PMIR1 were under high selection pressures during the divergence of cotton species. According to the studies in Arabidopsis, the Elongator components ELP1, ELP3, and ELP4 are responsible for the narrow leaf phenotype [52], and PMIR1 is involved in chloroplast and nuclear relocation in response to light [53]. However, the exact role of such genes in the success of cotton species adaptation requires further exploration.

## Conclusion

The present study provides a comprehensive analysis of transcriptomic content and gene expression patterns across different cotton species. It demonstrates the transcriptomic variation during species divergence within the genus Gossypium, offers further evidence to endorse the utilization of A_2_ as a substitute A-genome donor for analyzing expression changes in natural tetraploid cotton species, and uncovers biological pathways closely associated with cotton species adaptation. This work will be helpful for future understanding of the molecular mechanisms underlying variation of the leaf morphology and function during species divergence within the genus Gossypium.

## Material and methods

### Plant materials

The leaf transcriptome of the diploid A-genome cotton *G. herbaceum* (Zhongcao No.1) was generated by this work. The seeds were placed on the Hoagland’s media for germination and growth after removing the out shells. The four-week seedlings were then transferred to the pots (garden soil) and managed under natural conditions in the campus of Zhejiang Sci-Tech University (Hangzhou, China). The top three to five tender leaves below the apex were collected from the plants after a total of six-week growth. Three biological replicates were prepared for RNA sequencing (RNA-seq), with the leaves in each sample collected from at least five seedlings. The tissue samples were stored at -80 °C before use.

### RNA isolation, library construction, and sequencing

Total RNA was extracted using the Trizol reagent (Invitrogen, Carlsbad, CA, USA). The libraries were constructed and sequenced according to the manufacturer’s instructions (TruSeq RNA Sample Prep Kit, Illumina). In short, the mRNAs were purified using magnetic beads with oligo poly (T) attached. After fragmenting into about 300 bp, the mRNAs were converted into double-stranded cDNA. Three libraries were sequenced on the Illumina NovaSeq platform in Novogene (Beijing, China) with a pair-end strategy (2 × 150 bp). The generated RNA-seq data were deposited in the NCBI database Sequence Read Archive (SRA) (www.ncbi.nlm.nih.gov/sra) under the accession numbers SRR22211789-SRR22211791.

### Public data source

To investigate the gene expression in more cotton species, RNA-seq data of the seedling leaves of the outgroup *Theobroma cacao* and seven additional cotton species, *i.e.*, *G. arboreum* (A_2_), *G. thurberi* (D_1_), *G. raimondii* (D_5_), *G. stocksii* (E_1_), *G. australe* (G_2_), *G. hirsutum* (AD_1_), and *G. barbadence* (AD_2_), were collected from the public database SRA, with the accession numbers provided in Table 1. The reference genome sequences and annotation files were retrieved from CottonGen (www.cottongen.org) and MaGenDB (http://magen.whu.edu.cn/) [54, 55]. Please refer to the main text for the details of the data source.

### Construction of orthologous groups

OrthoFinder (version 2.5.4) was used to construct the orthologous groups (OGs) among investigated species with default parameters [56]. The subgenomes (A_t_ and D_t_) of the two tetraploid species AD_1_ and AD_2_ were considered independent organisms. For any species-complete OG that contained more than one gene copy from a single species, additional processing was performed to trim them into 1:1 (hereafter referred to as 1:1 OGs). First, all-to-all BLASTP was performed within each OGs, and the genes that showed more than 50% identity to the homologs in any other organisms were retained (E-value ≤ 1E-3 and coverage ≥ 50%) [57]. Then, we calculated the total bit scores of all possible 1:1 OGs and only the one with the highest bit score was further used [58]. Within a single 1:1 OG, the components from the subgenomes were considered homoeologs during expression change analysis.

### Estimation of gene expression levels

Quality control of the RNA-seq data was performed using Trimmomatic (version 0.33) (SLIDINGWINDOW:5:20 LEADING:3 TRAILING:3 MINLEN:36) [59]. The clean data was mapped to the reference genome using Hisat2 (2.2.1) [60]. The sorted bam files were generated using the scripts of Samtools (version 1.7) [59], and the gene expression levels (TPM, Transcripts Per Kilobase of exon model per Million mapped reads) were then estimated with the tool Stringtie2 (version 2.0.6) [61].

To assess the potential impact of sequencing depth on the number of expressed genes detected, we extracted 10% to 100% of the RNA-seq data (increases of 10% each time) for gene expression analysis with the aforementioned methods. For each depth, the number of expressed genes were determined by the average TPM ≥ 1 for the replicates of every organism, respectively. The accumulation curves were plot using ggplot2 (3.4.4) [62], and the *smooth* function was used to perform curve fitting with loess method.

### Clustering and principal component analyses

Based on the constructed 1:1 OGs and the estimated gene expression levels, we obtained an expression matrix consisting of the TPM values. To minimize the negative effect of background expression noise on correlation analysis, we filtered out the genes with an average TPM < 1 in all organisms. Then, the average TPM was log_2_-normalized after adding a pseudo count of 0.01. Pairwise Pearson correlation coefficients were subsequently calculated, and the principal component analysis (PCA) was conducted using the matrix of average TPM with the R package of *Hmisc* (version 5.0) and the function *prcomp* of R version 4.2.1, respectively.

### Gene expression evolution analyses

Based on the matrix of average TPMs, gene expression evolution within the genus Gossypium was analyzed using the TreeExp package (v2) according to its tutorial [63, 64]. The pairwise expression distances between any two organisms were calculated under the stationary Ornstein–Uhlenbeck (OU) model using the function *expdist*.

In addition, the genes within the constructed 1:1 OGs were classified into KEGG pathways according to the results from gene annotation. The pathways contained more than ten orthologs were used for next analysis. The relative rate of gene evolving for each pathway between Gossypium species was estimated using the function *RelaRate.test*. A *p*-value of 0.05 was used to indicate a significant difference between groups.

The strength of expression conservation for each gene was estimated based on Bayes’ theorem. Shortly, an inverse correlation matrix between designated species was first constructed with the function *corrMatInv* based on the TPM matrix. Then, the gamma distribution parameters were estimated with *estParaGamma*, and the gene-specific selection pressure was estimated by the functions *estParaQ* and *estParaWBayesian*.

To infer the transcriptome of the common ancestor (A_0_) of A_1_ and A_2_, an expression tree was first built based on the distance matrix function with the function *NJ*, with *T. cacao* used as the outgroup. The accuracy of the generated expression tree was estimated by bootstrap (500 replicates) using the function *boot.exphy*. To avoid the noise from polyploidy, only transcriptomes of diploids were used to estimate the gene expression levels in A_0_ with the function *aee*.

### Expression change analysis

Expression change upon polyploidization was estimated based on the dominant/additive ratio [65]. D_5_ was used as the D-genome ancestor, and A_1_, A_2_, and A_0_ were used as the A-genome ancestors in different tests. For the polyploid progenies AD_1_ and AD_2_, total expression of the A_t_ and D_t_ encoded homoeologs was used to represent the expression levels of orthologs to progenitors.

The dominant value (d) and additive value were calculated by P-(A + D)/2 and (A-D)/2, with the P, A, and D indicating the expression levels (average TPM) of orthologs in polyploids, A-genome ancestor, and D-genome ancestor, respectively. The gene expression patterns were classified according to the value of |d/a|, *i.e.*, additive (|d/a|≤ 0.2), partial dominance (0.2 <|d/a|≤ 0.8), dominance (0.8 <|d/a|≤ 1.2), and over dominance (d/a|> 1.2).

### Functional enrichment analysis

The orthologs from *T. cacao* represented the OGs during function analysis. The GO enrichment analysis was carried out with topGO via the online tools in MaGenDB.

### Supplementary Information

**Supplementary material 1.**

**Supplementary material 2.**

**Supplementary material 3.**

**Supplementary material 4.**

**Supplementary material 5.**

**Supplementary material 6.**

## Data Availability

RNA-seq generated by this work have been deposited in SRA database under the study SRP406534 with the accession numbers SRR22211789, SRR22211791, and SRR22211791 (https://trace.ncbi.nlm.nih.gov/Traces/?view=study&acc=SRP406534).

## Abbreviations

DGE: Differential gene expression
HEB: Homoeolog expression bias
ELD: Expression level dominance
OG: Orthologous groups
MPV: Mid-parent value
HSG/HLG: Genes under high/low selection pressure
