# Supplementary figures and images for: An insight into the gene expression evolution in Gossypium species based on the leaf transcriptomes
Source: BMC Genomics. 2024 Feb 14;25:179. doi: 10.1186/s12864-024-10091-x (PMC10868065; doi:10.1186/s12864-024-10091-x)

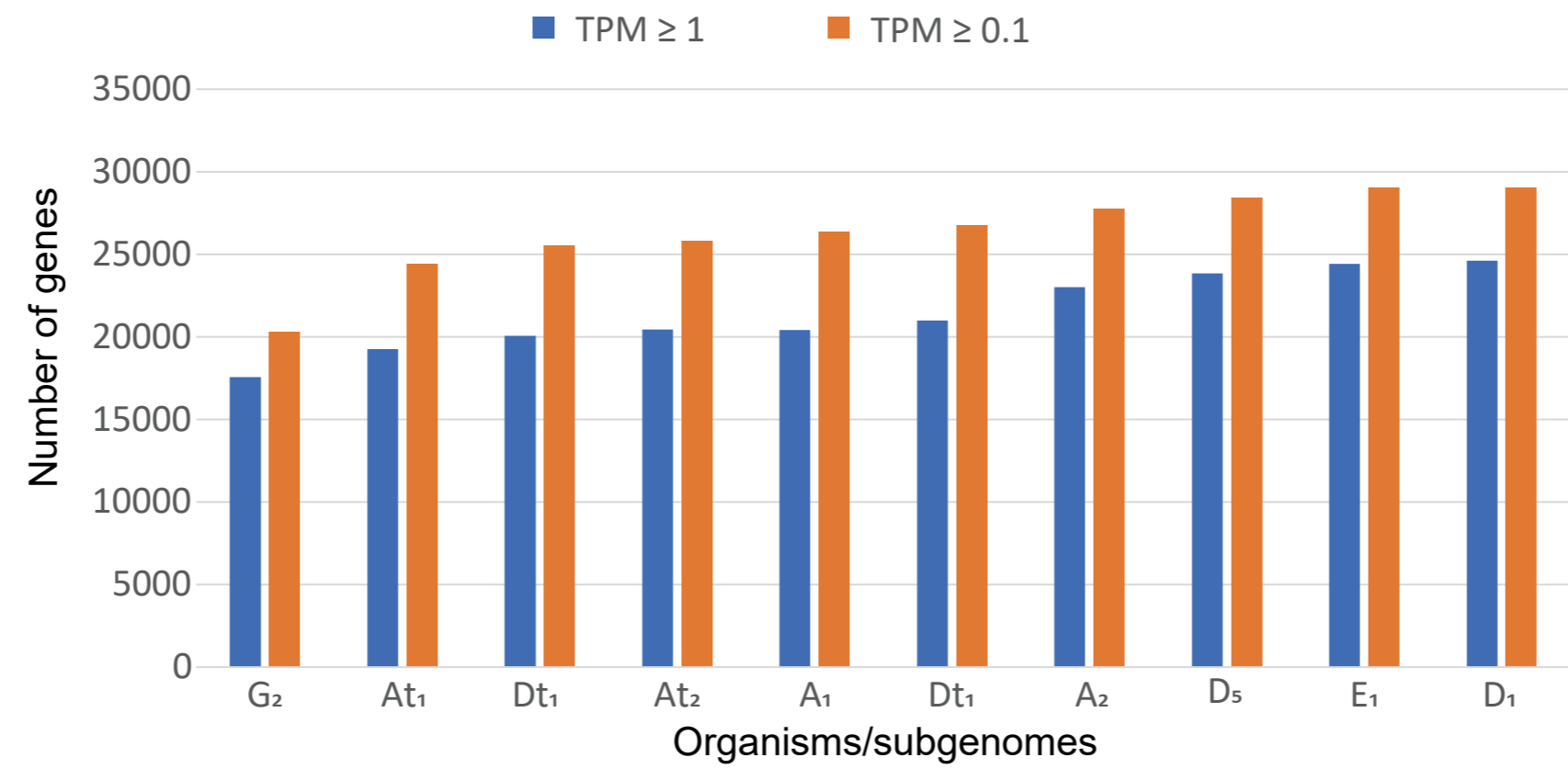

Supplement: Supplementary file 1 — Supplementary material 1. [file 12864_2024_10091_MOESM1_ESM.pdf]

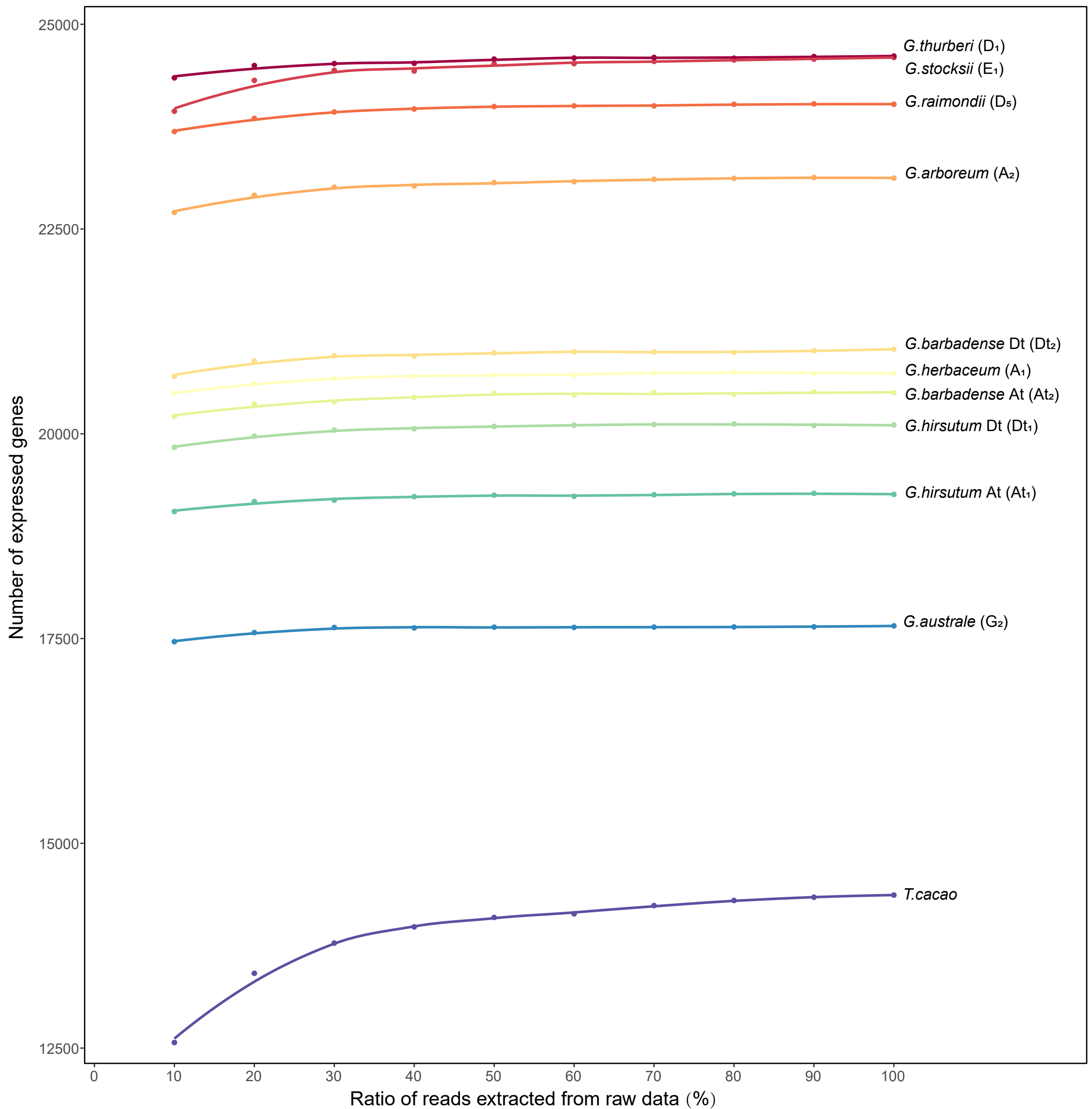

Supplement: Supplementary file 2 — Supplementary material 2. [file 12864_2024_10091_MOESM2_ESM.pdf]
